# Circulating CD4^+^CD28null T Cells May Increase the Risk of an Atherosclerotic Vascular Event Shortly after Kidney Transplantation

**DOI:** 10.1155/2013/841430

**Published:** 2013-10-01

**Authors:** Michiel G. H. Betjes, Willem Weimar, Nicolle H. R. Litjens

**Affiliations:** Erasmus Medical Center, Department of Internal Medicine, Division of Nephrology & Transplantation, D414, P.O. Box 2040, 3000 CA Rotterdam, The Netherlands

## Abstract

Proinflammatory CD4^+^ T cells without the costimulatory molecule CD28 (CD4^+^CD28null T cells) are expanded in patients with end-stage renal disease (ESRD) and associated with atherosclerotic vascular events (AVE). In a prospective study, the number of circulating CD4^+^CD28null T cells was established in 295 ESRD patients prior to receiving a kidney allograft. Within the first year after transplantation, an AVE occurred in 20 patients. Univariate analysis showed that besides a history of cardiovascular disease (CVDpos, HR 8.1, *P* < 0.001), age (HR 1.04, *P* = 0.02), dyslipidaemia (HR 8.8, *P* = 0.004), and the % of CD4^+^CD28null T cells (HR 1.04 per % increase, 95% CI 1.00–1.09, *P* = 0.01) were significantly associated with the occurrence of a posttransplantation AVE. In a multivariate analysis, only CVDpos remained a significant risk factor with a significant and positive interaction between the terms CVDpos and the % of CD4^+^CD28null T cells (HR 1.05, 95% CI 1.03–1.11, *P* < 0.001). Within the CVDpos group, the incidence of an AVE was 13% in the lowest tertile compared to 25% in the highest tertile of % of CD4^+^CD28null T cells. In conclusion, the presence of circulating CD4^+^CD28null T cells is associated with an increased risk for a cardiovascular event shortly after kidney transplantation.

## 1. Introduction

Patients with end-stage renal disease (ESRD) carry a highly increased risk for cardiovascular disease and an increased risk for an acute atherosclerotic vascular event shortly after kidney transplantation [1]. Traditional risk factors like smoking, hypertension, and hypercholesterolaemia can be identified but do not explain the full magnitude of the increment in risk [2–4]. In addition, treatment with statins has not resulted in a decreased cardiovascular mortality in ESRD or kidney transplant patients, indicating that other mechanisms of atherosclerotic disease are important [5–8]. Within the circulating CD4^+^ T cells population, a subset of cells can be identified that have lost the expression of the costimulatory molecule CD28 on their cell surface. These CD4^+^CD28null T cells are a rare population in most healthy individuals and usually do not exceed a few percent of the total CD4^+^ T cell population [9]. However, in patients with end-stage renal disease the numbers of circulating CD4^+^CD28null T cells may increase considerably and may represent >50% of the total CD4^+^ T cell population [10]. Phenotypical and functional analysis has identified these cells as highly differentiated proinflammatory T cells with intracellular granule containing cytotoxic molecules like granzyme and perforin [9]. Early observations showed a significant higher percentage of circulating CD4^+^CD28null T cells and perforin-expressing CD4^+^ T cells in non-ESRD patients with unstable angina. In a series of studies, it was subsequently shown that this type of CD4^+^ T cells is present in unstable atherosclerotic plaques and associated with an increased risk for recurrence of both acute coronary events and ischemic stroke [11–15]. In addition, human CD4^+^CD28null T cells were shown to invade and cause apoptosis of vascular smooth muscle cells in the atherosclerotic plaque of a human carotid artery xenotransplant in a mouse [16].

In accordance with these data, we found a significant correlation between the expansion of circulating CD4^+^CD28null T cells and atherosclerotic vascular events in a cross-sectional study including ESRD patients [17]. 

In this study, we investigated the hypothesis that the presence of circulating CD4^+^CD28null T cells establishes a risk factor for a novel atherosclerotic vascular event in patients in the first year after kidney transplantation.

## 2. Patients and Methods

### 2.1. Patient Population

Patients with ESRD, defined as a glomerular filtration rate of 15 mL/min or less with or without renal replacement therapy, were included the day before kidney transplantation. Patient data were collected in the period between June 2007 and June 2010. First and second generation immigrants from outside Western Europe were classified as being from non-Western European origin. All individuals included gave informed written consent, and the local medical ethical committee approved the study (MEC-2007-228). It was conducted according to the principles of the 2000 Declaration of Helsinki and Declaration of Istanbul 2008, and in compliance with International Conference on Harmonization/Good Clinical Practice regulations.

### 2.2. Clinical Evaluation

All patients referred to our out-patient clinic were screened for the presence of symptomatic coronary artery disease. Myocardial infarction (MI) reported in the medical history was confirmed if the medical record review demonstrated symptoms consistent with MI and the presence of either diagnostic ECG changes or cardiac enzymes. Evidence for cardiac ischaemia at the time of preoperative evaluation was obtained by graded exercise on a bicycle, or a dobutamine stress echocardiography was performed. These procedures were followed by coronary angiography when changes on the ECG were consistent with cardiac ischaemia. Signs of coronary atherosclerotic disease on coronary angiography were noted as either being absent or present. Symptomatic carotid artery disease was considered to be present when an atherosclerotic diseased carotid artery had been documented by angiography or ultrasonography, in most cases after a cerebrovascular accident. A cerebrovascular accident because of atherosclerosis was considered to be present if confirmed by a computed tomography scan of the brain. Atherosclerotic disease of the large peripheral arteries, including the aorta, was documented as being present if confirmed by angiography or computed tomography. The latter were performed because of symptoms of claudicatio intermittens, abnormalities noted on physical examination that indicated peripheral arterial stenosis or occlusion or aneurysmatic enlargement of the aorta. An atherosclerotic vascular event after kidney transplantation was defined as acute ischemia in any organ or limb caused by atherosclerotic disease. The events were categorized as cardiovascular, cerebrovascular, or peripheral vascular using similar criteria as stated above. In case of recurrent vascular events in the same patient, only the first event was recorded.

### 2.3. Risk Factors for Atherosclerotic Disease

Traditional risk factors for atherosclerotic disease considered in this study included age, male sex, smoking, diabetes, hyperlipidaemia, and hypertension. A history of past and current smoking was obtained. A patient who stopped smoking >10 years ago was considered not to have smoking as a risk factor. A patient was considered to have diabetes if he or she was taking insulin or oral hypoglycaemic agents or had previously received such treatment and was currently using dietary modification to control the condition. A patient was considered to have hyperlipidaemia if he or she had a serum cholesterol value of >6.5 mmol/L and/or was receiving antihyperlipidaemic treatment. A patient was considered to have hypertension if he or she had received the diagnosis or was being treated with antihypertensive medications at the time of evaluation. 

### 2.4. Identification of CD4^+^CD28null T Cells

Peripheral blood mononuclear cells were isolated from heparinized peripheral blood samples. The identification of T lymphocytes expressing CD4^+^ or CD8^+^ and lacking the CD28 molecule on the cell surface was done by FACS analysis, as has been described previously in detail [10]. The number of CD4^+^CD28null T cells was expressed as percentage of the total CD4^+^ T cells and as the total number of cells per mL blood. T cells were analyzed the day before kidney transplantation in all patients included. In 30 patients, the T cells were analyzed in the first year of transplantation.

### 2.5. Functional Analysis of CD4^+^CD28null T Cells

Unstimulated PBMC (2 × 10^6^/mL) were analyzed for cytotoxic potential by staining cell surface with AmCyan-labelled anti-CD3, PerCP-labelled anti-CD4 (BD, Erembodegem, Belgium), and FITC-labelled anti-CD28 (BD). Following fixation and permeabilization, cells were stained intracellularly with either one of the following phycoerythrin- (PE-) labelled antibodies directed against perforin (BD) or granzyme B (Sanquin, Amsterdam, The Netherlands). Percentages of granzyme B or perforin positive T cells were determined by analyzing the samples using the FACSCanto II (BD), selecting cells that have a typical lymphocyte scatter pattern, are CD4 positive, and either are positive or negative for cell surface CD28 expression. In order to analyze percentages of interferon-gamma (IFN-*γ*) or TNF-alpha (TNF-*α*) producing CD4^+^ T cells and that of CD4^+^ T cells expressing or lacking CD28, PBMC were stimulated with standard culture medium alone or combined with a mixture of PMA (50 ng/mL; Sigma Aldrich, St. Louis, MO, USA) and ionomycin (1 *μ*g/mL, Sigma Aldrich) for 6 hours in presence of the cytokine secretion inhibitor golgiplug (BD). Subsequently, the intracellular cytokine assay was performed as described recently in detail [18, 19]. Briefly, the cell surface of PBMC was stained with AmCyan-labelled CD3, Pacific Blue-labelled anti-CD4, and PerCP-Cy5.5-labelled anti-CD28 (all from BD). Following fixation and permeabilization, cells were stained with PE-labelled anti-TNF*α* (BD) and FITC-labelled anti-IFN-*γ* (BD). Percentages of cytokine positive CD4^+^ T cells were determined by analyzing the samples using the FACSCanto II (BD) and a similar gating approach as described above.

### 2.6. Statistical Analysis

All relevant variables were first examined in a univariate analysis for the relation with the occurrence of an atherosclerotic vascular event. To analyze the association with underlying renal disease, the patients were grouped into two categories: hypertensive nephropathy and primary renal disease. Differences between groups were analyzed with the student's *t*-test when the variable was normally distributed and otherwise by the Mann-Whitney test. Categorical data were analyzed by the *χ* ^2^ test. All statistical tests were two-sided. Variables with a *P* value below 0.05 were included in a proportional hazard regression analysis. The SPSS software version 10.1 was used for all statistical tests.

## 3. Results

### 3.1. Demographic and Clinical Characteristics

Two hundred and ninety patients were included in the study. The clinical characteristics are shown in Table 1 and reflect the high burden of atherosclerotic disease in this group of patients, as more than a third of all patients had a medical history of atherosclerotic disease. In 20 patients, an atherosclerotic vascular event was recorded after kidney transplantation. Within these 20 patients, 16 cardiovascular events, 3 cerebrovascular and 1 peripheral-vascular occurred. The median time from transplantation to event was 8 days, and 80% of all events occurred within 3 months after surgery.

Patients with an atherosclerotic vascular event (event group) differed significantly from the patients without an event in the first year after transplantation (no-event group). The event group showed a higher median age (62 versus 52 years), a threefold increased prevalence of a history of atherosclerotic disease (25% versus 75%), and a twofold increased prevalence in dyslipidemia (45% versus 90%) without other differences in classical risk factors for atherosclerosis. Five patients in the event group had no history of atherosclerotic vascular disease (2.3% of all patients without known vascular disease), while 15 patients in the event group were known with atherosclerotic vascular disease (18.3% of all patients with known vascular disease).

### 3.2. Circulating CD4^+^CD28null T Cells Are a Risk Factor for an Atherosclerotic Vascular Event

**Figure 1:**
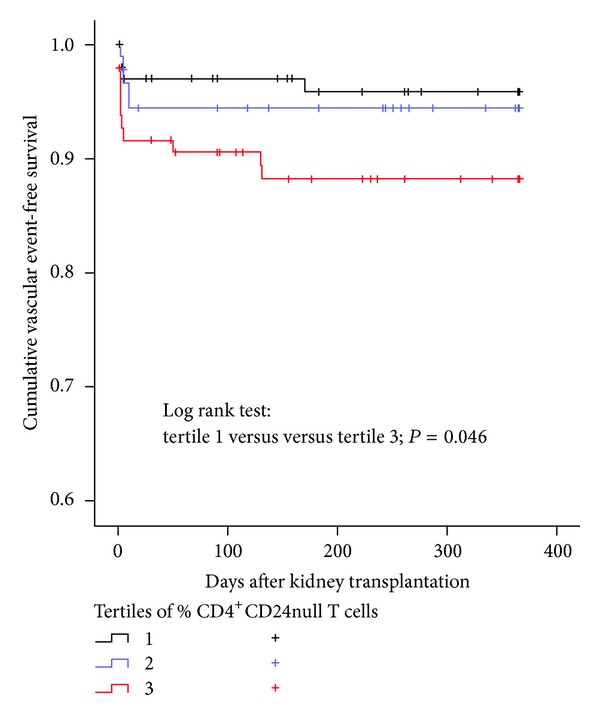
Kaplan-Meier vascular event-free survival after kidney transplantation stratified per tertile of percentage CD4^+^CD28null T cells in 290 kidney transplant patients. Range of percentage of CD4^+^CD28null T cells in tertile 1: 0.00–0.30%, tertile 2: 0.31–5.16%, and tertile 3: 5.17–63.11%.

Prior to kidney transplantation, the average percentage (11.7% versus 5.8%, *P* = 0.002) of circulating CD4^+^CD28null T cells was increased in patients in the event group compared to patients in the no-event group. Although the average absolute number of CD4^+^CD28null T cells was also increased in patients in the event group, this difference did not reach statistical significance (38 versus 25 × 10^4^/mL, *P* = 0.23). A Kaplan-Meier analysis of vascular event-free survival was performed by categorizing patients within tertiles of CD4^+^CD28null T cells. A significant difference (log-rank test, *P* = 0.046) in event-free survival was observed between patient groups having the lowest (tertile 1, event-free survival at 1-year posttransplantation 96%) and highest (tertile 3, event-free survival at 1-year posttransplantation 88.5%) percentages of circulating CD4^+^CD28null T cells (Figure 1).

The percentage of circulating CD4^+^CD28null T cells was included as a covariate in a proportional hazard regression analysis (Table 2). Univariate analysis showed that besides known atherosclerotic disease (HR 8.1, *P* < 0.001), age (HR 1.04, *P* = 0.02), dyslipidaemia (HR 8.8, *P* = 0.004), and the % of CD4^+^CD28null T cells (1.04 per % increase, *P* = 0.01) were significantly associated with the occurrence of a posttransplantation atherosclerotic vascular event. In a multivariate analysis, only known atherosclerotic disease remained a significant risk factor (HR 16, *P* < 0.01), and % of CD4^+^CD28null T cells reached a *P* value of 0.05. A significant and positive interaction existed between the terms “known atherosclerotic disease” and “% of CD4^+^CD28null T cells” (HR 1.05, *P* < 0.001). 

**Figure 2:**
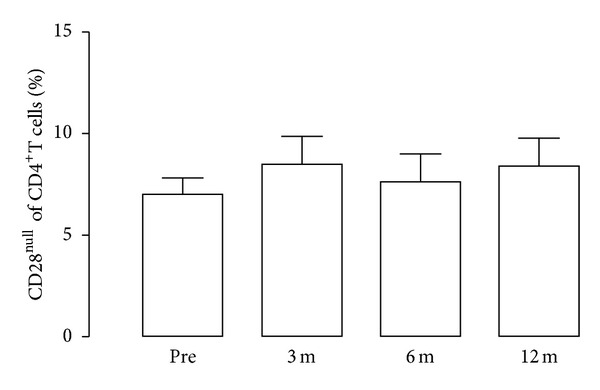
The percentage of CD4^+^CD28null T cells after kidney transplantation. On the *y*-axis the percentage of CD4^+^CD28null cells is given in relation to time after kidney transplantation (given in months) and before transplantation (pre) in 30 patients.

The number of CD4^+^CD28null T cells was recorded at regular intervals after kidney transplantation but did not change over time (Figure 2).

### 3.3. Circulating CD4^+^CD28null T Cells Have Similar Cytotoxic and Proinflammatory Potential in Patients with or without a History of Atherosclerotic Disease

**Figure 3:**
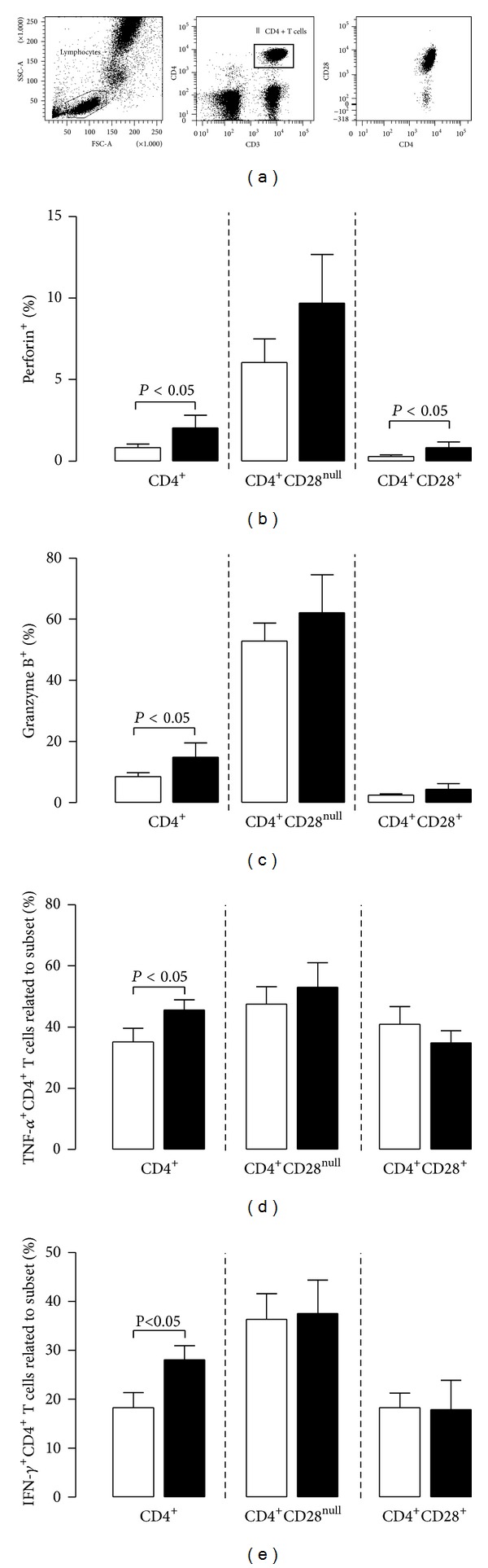
Expression of cytotoxic molecules and proinflammatory cytokines in the total population of circulating CD4^+^ T cells and the CD28^+^ and CD28null subsets within ESRD patients before kidney transplantation. In (a), a typical flow cytometric example is depicted with respect to the dissection of CD4^+^ T cells into those expressing CD28 (CD4^+^CD28^+^) and those lacking CD28 (CD4^+^CD28null). Next, we determined the cytotoxic potential by analyzing percentages of perforin^+^ (b) and Granzyme B^+^ (c) CD4^+^ T cells as well as those expressing or lacking CD28 in 11 ESRD patients known with atherosclerotic disease (CVDpos, closed bars) before transplantation and compared that to 27 age- and sex-matched ESRD patients, not known with preexisting atherosclerotic disease (CVDneg, open bars). In addition, PBMC of 8 CVDpos ESRD patients (closed bars) and 12 age- and sex-matched CVDneg patients (open bars) were stimulated with PMA and ionomycin in order to be able to analyze percentages of the proinflammatory cytokines TNF-*α* ^+^ (d) as well as IFN-*γ* ^+^ (e) within CD4^+^ and CD4^+^CD28^+^ and CD4^+^CD28null T cells.

Next, we investigated whether CD4^+^CD28null T cells are functionally different between patients with or without a medical history of atherosclerotic disease (Figure 3). As shown in the top panel of Figure 2, CD4^+^CD28null T cells can be easily identified as there is a clear distinction between CD28^+^ and CD28null CD4^+^T cells (a). Within the total patient population, perforin (b) and granzyme B (c) positive CD4^+^ T cells were higher (*P* < 0.0001) within the CD28null fraction when compared to CD28^+^ fraction; that is, percentages amounted to 7.65 ± 1.26% versus 0.59 ± 0.12% for perforin and 64.79 ± 3.79% versus 5.18 ± 1.30% for granzyme B, within CD28null and CD28^+^CD4^+^ T cells, respectively.

After polyclonal stimulation, the percentages of TNF*α* (d) and IFN-*γ* (e) positive CD4^+^CD28null T cells were higher compared to CD4^+^CD28^+^ T cells (49.72 ± 4.58% versus 38.48 ± 2.79% for TNF*α*, *P* < 0.05 and 36.81 ± 4.07% versus 18.09 ± 2.91% for IFN-*γ*, *P* < 0.01), but no differences were found between CVDpos (closed bars) and CVDneg (open bars) ESRD patients with respect to the cytotoxic or proinflammatory potential of CD4^+^CD28null T cells. 

In previous studies [10, 20], we have repeatedly shown that not only the numbers of CD4^+^CD28null T cells are increased in ESRD patients but also their expression levels of cytotoxic molecules and proinflammatory molecules, when compared to data of healthy individuals.

## 4. Discussion

In recent years, a number of studies in the field of cardiology have been published which showed that the population of circulating CD4^+^CD28null T cells is relatively expanded in patients with atherosclerotic disease and associated with recurrence of a cardiovascular event [9, 15]. Subsequently, a possible pathogenetic mechanism was identified, as this particular cell population is capable of infiltrating and destabilizing atherosclerotic plaques [16]. The findings of this study indicate that an expanded CD4^+^CD28null T cell population may also act as a nontraditional risk factor for a vascular event in ESRD patients receiving a kidney transplant. In accordance with previous published studies, we documented that the risk for a vascular event was specifically increased within the first 3 months after kidney transplantation [21, 22].

The CD4^+^CD28null T cells constitute a remarkable subpopulation of CD4^+^ T cells as they are proinflammatory and cytotoxic in nature and carry molecules on their cell surface which are normally found on natural killer cells [23]. Their lack of chemokine receptor CCR7 expression precludes migration to lymphatic tissues, but instead they can respond to fractalkine, a chemokine which is produced in the diseased vascular wall [10, 24, 25]. Therefore, these cells are very well equipped for migration into inflammatory tissue such as atherosclerotic plaques where they may increase plaque instability with subsequent plaque rupture. It is unknown whether there is true antigen-specific responsiveness involved as these cells can also be found within inflammatory tissue of the joint, gut or muscle in unrelated diseases like rheumatoid arthritis, colitis, and dermatomyositis [25–27]. Cytomegalovirus infection is associated with expansion of these cells, but only a minority of CD4^+^CD28null T cells could be identified as specific for cytomegalovirus antigen [10, 28]. Others have documented heat shock protein-60 antigen specificity within the CD4^+^CD28null T cell population which may in part explain their presence in a variety of different inflamed tissues [13, 29]. Of note, the expansion of CD4^+^CD28null T cells is a common finding in many unrelated proinflammatory conditions like ESRD, HIV, infection and autoimmune diseases [23]. Therefore, it may represent a nonclassical risk factor for atherosclerotic disease particularly in inflammatory patient populations which are known for their increased risk for atherosclerosis [30–33]. CD4^+^CD28null T cells have not only been implicated in atherosclerotic plaque rupture but also in early stages of atherosclerosis such as arterial media thickening [34]. Therefore, expanded numbers of CD4^+^CD28null T cells may both promote the initiation of atherosclerotic disease and inflammation of an already established atherosclerotic plaque. Data from this study are supportive of such a hypothesis although the multivariate analysis indicated that the presence of preexistent CVD was by far the most dominant risk factor for a new AVE after transplantation.

Of particular interest, a significant interaction existed between a history of atherosclerotic disease and increased frequencies of CD4^+^CD28null T cells for the risk of a de novo vascular event after kidney transplantation. This observation fits in the concept that destabilization of pre-existent vulnerable nonobstructive plaques is the leading cause of a postoperative atherosclerotic event [35, 36]. These plaques are more common in patients known with a medical history of atherosclerotic disease but because of their nonobstructive nature are not causing symptomatic ischaemia. The explanation for atherosclerotic plaque destabilization in this setting includes temporarily upregulation of plaque inflammation with promotion of thrombus formation on the plaque surface by activation of the coagulation or complement system [37, 38]. Whether CD4^+^CD28null T cells already present within the plaque [15] become activated or are newly recruited from the circulation is not known. We tested whether CD4^+^CD28null T cells from patients with or without pre-existent CVD differ in their functional capacity but could not find significant differences. In previous studies, we did show an increased proinflammatory profile of these cells from ESRD patients compared to healthy individuals [10, 20], indicating that CD4^+^CD28null T cells from ESRD patients may have an increased propensity for causing atherosclerotic plaque rupture. The results of the present study showed a tendency for higher absolute numbers of CD4^+^CD28null T cells in patients with a posttransplant AVE, but statistical significance was not reached. All previous studies on this subject did not measure total number of CD4^+^CD28null T cells. However, similar to our results they found significant relations between the percentage of CD4^+^CD28null T cells among the total circulating CD4^+^ T cell population and the risk for a cardiovascular event [11, 12, 14, 15]. This finding might implicate that the relative number of CD4^+^CD28null T cells among the total T cell population is of more importance than absolute numbers. However, current data are insufficient to back up this hypothesis and further research is needed to elucidate more fully the mechanisms involved in postoperative destabilization of atherosclerotic plaques by CD4^+^CD28null T cells. Interestingly, the number of CD4^+^CD28null T cells does not change after kidney transplantation and therefore may remain a nonclassical cardiovascular risk factor in transplanted patients.

Given the role of inflammation in atherosclerotic plaque destabilization, immune suppressive medication like mycophenolate mofetil may counteract the increased postoperative risk for vascular events [39]. In this study, patients were given high dose steroids and mycophenolate mofetil shortly after transplantation, but whether this has influenced or prevented formation of unstable plaques cannot be concluded from our data. A possible role for anti-inflammatory therapy was shown as the use of anti-TNF receptor monoclonal antibodies decreased the frequency of CD4^+^CD28null T cells [40]. Also, the use of statins in patients with cardiovascular disease was associated with both a lowered C-reactive protein and a reduced frequency of circulating of CD4^+^CD28null T cells [41]. CD4^+^ regulatory T cells may be pivotal in controlling atherosclerotic plaque inflammation, but CD4^+^CD28null T cells are unfortunately relatively insensitive for their suppressive effects [42, 43]. At present, there are no studies performed which have assessed the efficacy of interventions aimed at reducing the pathogenic role of proinflammatory CD4^+^CD28null T cells in atherosclerosis.

In conclusion, a higher frequency of circulating CD4^+^CD28null T cells is associated with an increased risk for an atherosclerotic vascular event shortly after kidney transplantation, particularly in patients known with atherosclerotic vascular disease. Given the proinflammatory and plaque destabilization potential of these cells, their direct involvement in plaque rupture seems likely.

## Figures and Tables

**Table 1:** Demographic and clinical characteristics of patients receiving a kidney allograft with or without an atherosclerotic vascular event in the first year after transplantation.

	Atherosclerotic event in the first year after kidney transplantation		*P* value
	No	Yes	
Number of patients	270	20	
Age (median and IQR in years)	52 (40–62)	62 (47–67)	0.02
Male gender	69.3%	75%	0.59
Non-Western European origin	27.6%	35%	0.48
CMV seropositive	103/167	3/17	0.052
Cumulative years on dialysis treatment (median and interquartile range)	1.4 (0–3)	1.7 (1–4)	0.49
History of atherosclerotic disease	24.3%	75%	<0.001
Cardiovascular	14.9%	52.6%	<0.001
Cerebrovascular	7.9%	25%	0.02
Aorta/peripheral arteries	6.7%	15.8%	0.15
Underlying kidney disease			0.61
Hypertensive nephropathy	30.2%	42.1%	
Primary glomerulopathy	24.3%	21.1%	
Diabetic nephropathy	10.8%	5.3%	
Polycystic kidney disease	11.9%	21.1%	
Other	16.0%	10.5%	
Unknown	6.7%	0.0%	
Prevalence of risk factors for atherosclerosis			
Hypertension	87.1%	94.4%	0.36
Hyperlipidaemia	43.7%	90.9%	<0.001
Smoking	33.6%	50.0%	0.18
Diabetes mellitus	22.1%	31.6%	0.58
% CD4^+^CD28null T cells (mean ± SD)	5.8 ± 9.6%	11.7 ± 13.5%	0.01
CD4^+^CD28null T cell × 10^4^/mL (mean ± SD)	25.9 ± 43.4	38.4 ± 46.7	0.23

**Table 2:** Univariate and multivariate analysis of clinical and demographic parameters in association with the incidence of an atherosclerotic vascular event within the first year after kidney transplantation.

	Univariate analysis	Multivariate analysis	Hazard ratio (95% confidence interval)
	*P* value	*P* value	
Male gender	0.56	0.13	—
Age (years)	0.02	0.61	1.01 (0.95–1.07)
Years of dialysis treatment	0.51	0.42	—
Non-Western European origin	0.47	0.83	—
History of atherosclerotic disease	<0.001	0.01	13.9 (1.7–113.5)
Diabetes mellitus	0.53	0.75	—
Smoking	0.19	0.22	—
Dyslipidemia	0.01	0.12	4.7 (1.71–13.22)
Hypertension	0.38	0.44	—
% CD4^+^CD28null T cells	0.01	0.05	1.04 (1.00–1.09)
% CD4^+^CD28null T cells^∗ ^ history of atherosclerotic disease	—	0.001	1.07 (1.03–1.11)

*Interaction between the terms % CD4^+^CD28null T cells and a history of atherosclerotic disease on the incidence of an atherosclerotic event after kidney transplantation.
